# Long-read, chromosome-scale assembly of *Vitis rotundifolia* cv. Carlos and its unique resistance to *Xylella fastidiosa* subsp. *fastidiosa*

**DOI:** 10.1186/s12864-023-09514-y

**Published:** 2023-07-20

**Authors:** Matthew Huff, Amanda M. Hulse-Kemp, Brian E Scheffler, Ramey C Youngblood, Sheron A Simpson, Ebrahiem Babiker, Margaret Staton

**Affiliations:** 1 Department of Entomology and Plant Pathology, University of Tennessee, Knoxville, TN 37996 USA; 2 Genomics and Bioinformatics Research Unit, USDA-ARS, Raleigh, NC 27606 USA; 3 Department of Crop and Soil Sciences, North Carolina State University, Raleigh, NC 27606 USA; 4 Genomics and Bioinformatics Research Unit, USDA-ARS, Stoneville, MS 38776 USA; 5 Institute for Genomics, Biocomputing and Biotechnology, Mississippi State University, Starkville, MS 39762 USA; 6 USDA-ARS Thad Cochran Southern Horticultural Laboratory, Poplarville, MS 39470 USA

**Keywords:** Muscadine grape, Grape, Pangenome, Transcriptome, Pierce’s disease

## Abstract

**Background:**

Muscadine grape (*Vitis rotundifolia*) is resistant to many of the pathogens that negatively impact the production of common grape (*V. vinifera*), including the bacterial pathogen *Xylella fastidiosa* subsp. *fastidiosa* (*Xfsf*), which causes Pierce’s Disease (PD). Previous studies in common grape have indicated *Xfsf* delays host immune response with a complex O-chain antigen produced by the *wzy* gene. Muscadine cultivars range from tolerant to completely resistant to *Xfsf*, but the mechanism is unknown.

**Results:**

We assembled and annotated a new, long-read genome assembly for ‘Carlos’, a cultivar of muscadine that exhibits tolerance, to build upon the existing genetic resources available for muscadine. We used these resources to construct an initial pan-genome for three cultivars of muscadine and one cultivar of common grape. This pan-genome contains a total of 34,970 synteny-constrained entries containing genes of similar structure. Comparison of resistance gene content between the ‘Carlos’ and common grape genomes indicates an expansion of resistance (R) genes in ‘Carlos.’ We further identified genes involved in *Xfsf* response by transcriptome sequencing ‘Carlos’ plants inoculated with *Xfsf*. We observed 234 differentially expressed genes with functions related to lipid catabolism, oxidation-reduction signaling, and abscisic acid (ABA) signaling as well as seven R genes. Leveraging public data from previous experiments of common grape inoculated with *Xfsf*, we determined that most differentially expressed genes in the muscadine response were not found in common grape, and three of the R genes identified as differentially expressed in muscadine do not have an ortholog in the common grape genome.

**Conclusions:**

Our results support the utility of a pan-genome approach to identify candidate genes for traits of interest, particularly disease resistance to *Xfsf*, within and between muscadine and common grape.

**Supplementary Information:**

The online version contains supplementary material available at 10.1186/s12864-023-09514-y.

## Background

Muscadine (*Vitis rotundifolia* Michx., syn. *Muscadinia rotundifolia* 2*n* = 2*x* = 40) is a species of grape native to the southeastern United States (Supplemental Figure S1). It is part of the *Muscadinia* subgenus of *Vitis*, which contains only three species (Hickey et al., 2019). In comparison, the subgenus *Euvitis* (bunch grapes) exhibits greater diversity and includes about 60 species. Compared to the common table grape (*V. vinifera*; a member of the *Euvitis* subgenus), muscadine grapes are a small-market crop [1], but they are also more pest and drought tolerant [2]. Based on phylogenetic evidence, the *Vitis* genus originated in North America 28 million years ago, and the *Muscadinia* subgenus diverged from *Euvitis* approximately 18 million years ago [3]. Native Americans used the muscadine fruit both as a food source and a dye prior to European colonization, and wineries in the Southeast have produced special red and white wines from muscadine berries since the 16th century [4]. Muscadine grapes have increased in commercial interest outside of culinary applications, including medicinal use and nutritional supplements, due to higher antioxidant content and an increased diversity of bioactive compounds relative to other grape species. Several studies demonstrate treatment of cancer cells in vitro with muscadine grape skin extracts (MGSEs) has been found to increase cytotoxicity and trigger apoptosis in breast cancer cells [5, 6] and prostate cancer cells [7]. In the context of grape breeding, muscadines have attracted interest in providing a resource for beneficial traits to be introduced into bunch grape species. However, breeding efforts are complicated by the different number of chromosomes caused by fusion of two muscadine chromosomes in the *Vitis vinifera* genome [8, 9].

Pierce’s Disease (PD) has become a major limiting factor in the production of wine and table grapes in southern California, the center of winemaking in the continental United States (US). Symptoms include leaf necrosis, leaf scorch, and dieback, leading to vine death within three to five years (Supplemental Figure S2). PD is caused by the xylem-limited bacterial pathogen *Xylella fastidiosa* subsp. *fastidiosa* (*Xfsf*) and is spread by xylem-feeding sharpshooter family (Cicadelladea) insect vectors [10]. Disease spread was limited by short flying distance and low feeding rate of the insect vector, however, the introduction of a more efficient vector in the 1990s, the glassy-winged sharpshooter (*Homalodisca vitripennis*), exacerbated the impact of PD in California [11]. *Xfsf* is a gram-negative bacterium that is indigenous to the Gulf Coast region of the US, but it has since spread to other regions of both American continents as an invasive disease [12]. The bacteria clog the xylem vessels of susceptible grape genotypes, leading to water and nutrient stress. PD symptoms usually appear in late summer during high temperatures or when the plants are exposed to drought conditions. In most muscadine grapes, PD is of little concern, with disease symptoms typically limited to occasional necrosis or marginal leaf burn on tolerant cultivars (Supplemental Figure S1).

Previous studies have found clues to the physiology and molecular basis of resistance to PD in both muscadines and common grapes. In comparison to susceptible bunch grapevines, tolerant muscadine cultivars infected with *Xfsf* produced higher frequencies of tyloses and gums, which occlude xylem vessels to encapsulate and prevent internal spread of the bacteria [13]. In addition, gums and tannins occur more frequently in the PD-resistant muscadine grape cultivar ‘Noble’ after PD inoculation than in the PD-tolerant cultivar ‘Carlos’ [14, 15]. Furthermore, higher amounts of xylem sap β-1,3-glucanase and peroxidase was observed in PD-tolerant muscadine grapes compared to PD-susceptible bunch grapes [16]. In PD, knockout of the *wzy* gene in *Xfsf* was observed to result in a tolerant host response [17]. The *wzy* mutants were unable to produce a complex lipopolysaccharide O-antigen that, in wild-type *Xfsf*, delays host immune response. Comparison of infected and uninfected grape vines (var. Thompson Seedless) found an up-regulation of antioxidant strategies, cell wall modification enzymes, and pathogenesis-related proteins [18]. Although structural barriers provide some degree of resistance/tolerance against pathogens, little is known about the relationship between muscadine grape anatomy and its tolerance to *Xfsf*.

At present, two muscadine genomes have been published. The first genome was a haplotype-phased, chromosome-level assembly of the male cultivar ‘Trayshed,’ representing about 86.2% of the estimated 484.3 Mbp genome size [19]. The genome was used to verify the fusion of muscadine chromosome 7 and 20 in common grape as chromosome 7, previously only supported by muscadine linkage group evidence [20, 21]. Compared to the assembly of Cabernet Sauvignon, a disease-susceptible cultivar of bunch grape, the ‘Trayshed’ assembly had an expansion of Toll/Interleukin-1 Receptor-like Nucleotide-Binding Site Leucine-Rich Repeat (TIR-NBS-LRR) proteins, a class of disease resistance genes. A second assembly was recently published for the ‘Noble’ cultivar and represents 81.5% of the estimated total genome size [22]. Additionally this study included a genome-wide association study (GWAS) which identified 52 quantitative trait nucleotides (QTNs) associated with 12 berry-related traits. Due to the similarity between *Muscadinia* and *Euvitis* genomes, these QTN markers can be used to study comparative genetics between species. As the dynamic of genomics shifts towards a pan-genome model, which covers genetic content across multiple cultivars of a species, these two assemblies can be used together to provide a more accurate understanding of the relationship between different cultivars [23, 24].

To examine gene-expression changes in response to inoculation with *Xfsf*, RNA-Seq was performed on the muscadine ‘Carlos,’ a bronze-skinned, commercial cultivar. Unlike most muscadine cultivars with PD resistance, Carlos is considered tolerant and may show symptoms [14]. ‘Carlos’ is the predominant bronze-skinned juice cultivar and is generally selected for its high yield and dry stem scars. A ‘Carlos’ reference genome was constructed to improve the accuracy of the RNA-Seq data analysis and expand existing muscadine genomic resources. To establish a genomic framework for this comparison within and across species, we developed a pan-genome resource incorporating three muscadine genome assemblies and a common grape genome assembly. To further understand the differences in response between muscadine and common grapes, we compared our gene expression data to grapes inoculated with both wild-type *Xfsf* and *wzy* knock-out mutants [17].

## Results

I. Genome Assembly Information and Statistics.

Our ‘Carlos’ chromosome-scale assembly utilized PacBio CLR long-read sequencing to generate DNA scaffolds. The initial assembly consisted of 384 contigs arranged into 355 scaffolds with a total assembly size of 413 Mb and a contig N50 of 2.3 Mb (Supplemental Table S1). Reference-guided scaffolding of the initial ‘Carlos’ assembly to the ‘Trayshed’ haplotype 1 assembly [19] arranged the contigs into 76 scaffolds, including 20 chromosome-level scaffolds that contained 99% of the sequenced bases. After resolving gaps, the assembly consisted of 112 contigs in 76 scaffolds with a low percentage of uncalled bases (Table 1). The final assembly has a total sequence length of 413 Mb with the 20 assembled chromosomes representing 97.7% of the total length. Assessment of our assembly by benchmarking universal single-copy orthologs (BUSCOs) indicated the sequence was largely complete [25]. This assembly is slightly longer than the 394 Mb for ‘Noble’ and 400 Mb for ‘Trayshed’, but all three are notably lower than the flow cytometry estimate of 483 Mb, suggesting the flow cytometry is likely an overestimate [19, 22].

**Table 1:** Summary of the Vitis rotundifolia ‘Carlos’ genome assembly

	‘Carlos’ (All Scaffolds)	‘Carlos’ (Chromosomes Only)	Trayshed (v2.0, hap 1)	Noble
Total Scaffold Length	413,913,702	404,563,012	400,450,509	393,820,999
Total Contig Length	413,908,995	404,559,443	399,804,757	393,085,488
No. of Scaffolds	76	20	20	20
No. of Contigs	112	55	781	7,797
Largest Scaffold	31,234,097	31,234,097	34,098,201	30,244,861
Smallest Scaffold	2,758	13,845,986	14,236,136	11,958,844
# of Ns	3,707	3,569	645,752	735,511
GC (%)	34.11	34.01	33.76%	33.54%
Protein Coding Genes	27,923	27,747	25,706	26,394
Total Transcripts (w/Isoforms)	40,416	40,219	41,518	26,394
Busco description	Number in genome			
Complete BUSCOs (C)	1583 (97.9%)		1522 (94.3%)	1579 (97.9%)
Complete, Single-Copy (S)	1556 (96.4%)		1477 (10.5%)	1541 (95.5%)
Complete, Duplicated (D)	25 (1.5%)		45 (2.8%)	38 (2.4%)
Fragmented BUSCOs (F)	21 (1.3%)		24 (1.5%)	25 (1.5%)
Missing BUSCOs (M)	12 (0.8%)		68 (4.2%)	10 (0.6%)
Total	1614		1614	1614

*BUSCO scores were identical when considering all scaffolds and only chromosomes

II. Gene Annotation Report.

**Fig. 1:**
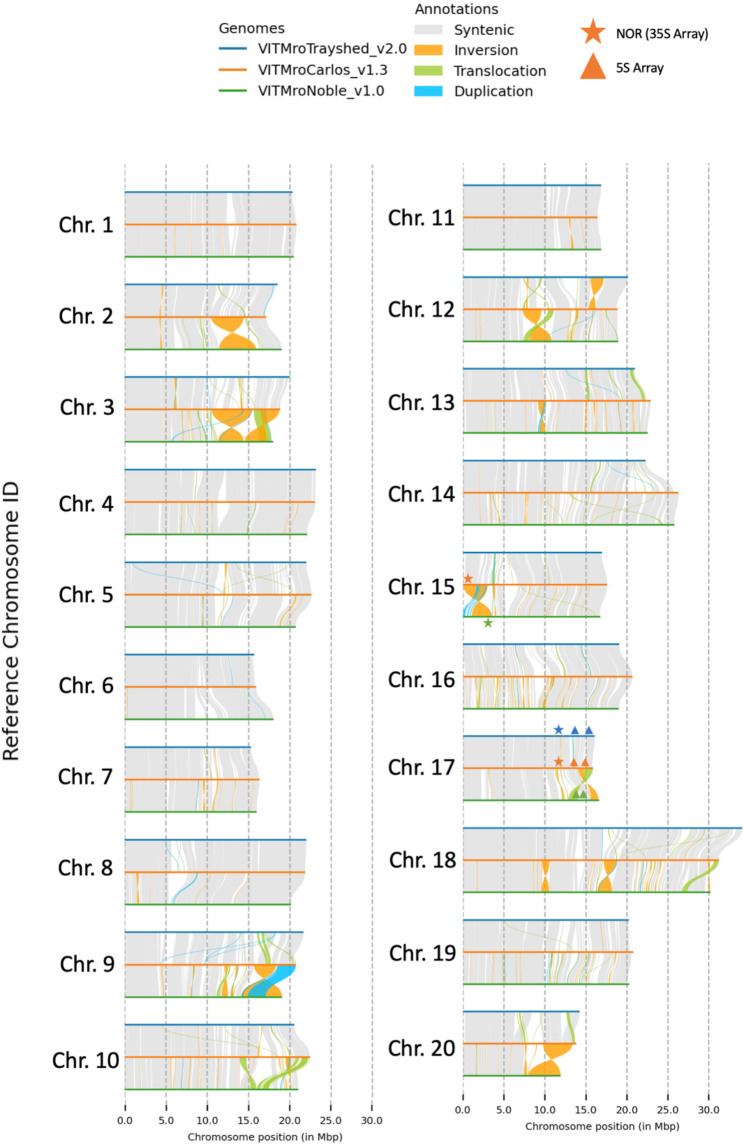
Plot of collinearity between *Vitis rotundifolia*‘Carlos’, *V. rotundifolia* ‘Trayshed’, and *V. rotundifolia* ‘Noble’ assemblies. The location of rDNA containing a nucleolus organizing region (NOR) is denoted by a star, while rDNA containing a 5 S array is denoted by a triangle. Symbols are correlated by color to the associated cultivar (see figure legend)

Using a combination of *de novo* repeat calling and known plant repeats, 51.12% of the ‘Carlos’ assembly was identified as repetitive (Supplemental Table S2). Long-terminal repeats (LTRs) were the most common repeats (15.96%), primarily consisting of Gypsy/DIRS1 (8.06%) and Ty1-Copia (7.33%) retrotransposon families. A total of 27,923 genes were identified in the ‘Carlos’ assembly, of which 27,747 (99.4%) of these genes were located on the 20 assembled chromosomes. The inclusion of RNA-Seq alignment data allowed GeMoMa to identify 40,219 gene isoforms [26], and tRNAscan identified 611 tRNAs across all 20 chromosomes [27]. RNAmmer identified two nucleolus organizing regions (NORs) with 18 S-5.8 S-26 S (35 S) rDNA arrays on chromosomes 15 and 17, as well as two distinct 5 S rDNA regions on chromosome 17 (Fig. 1) [28].

III. Comparison of assembly to existing cultivars and common grape genome.

Overall chromosome size was consistent among all three assemblies, though chromosome 18 was notably longer in the ‘Trayshed’ assembly than in the other cultivar assemblies. Alignment of the ‘Carlos’ assembly to the existing ‘Trayshed’ and ‘Noble’ assemblies demonstrated an overall similar structure among cultivars (Fig. 1) [19, 22]. Most regions in all three genomes were syntenic, with 83.8% of the ‘Carlos’ genome syntenic with the ‘Trayshed’ assembly and 73.4% syntenic with the ‘Noble’ assembly (Table 2). In these syntenic comparisons with ‘Carlos’, ‘Trayshed’ had a higher number of sites with translocations compared to ‘Noble’. In contrast, the ‘Noble’ genome assembly had a larger number of inversions despite containing more syntenic regions with ‘Carlos’. Chromosome 8 in the ‘Carlos’ assembly had a gap in synteny in its alignments to both ‘Trayshed’ and ‘Noble’ at approximately 5 Mb.

**Fig. 2:**
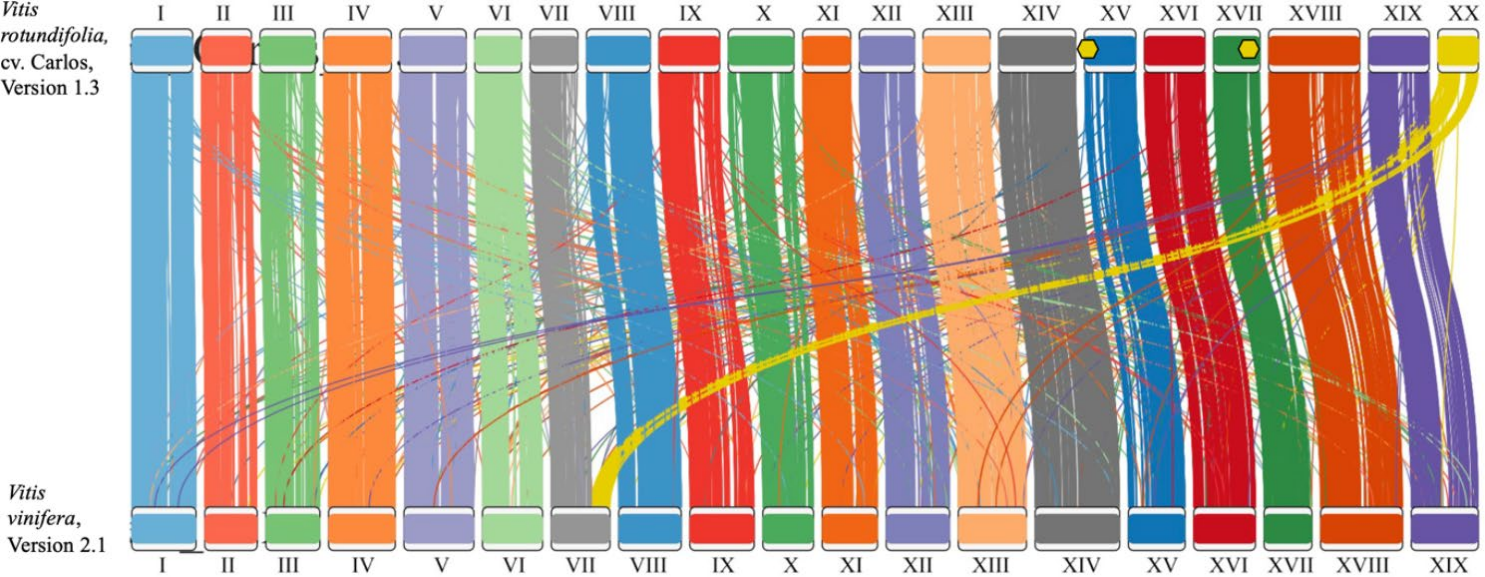
**Plot of collinearity between*Vitis rotundifolia*‘Carlos’ and*Vitis vinifera*v2.1 assemblies.** Figure generated using RIdeogram version 0.2.2. Yellow hexagons indicate the location of 35 S rDNA arrays

Alignment of the ‘Carlos’ cultivar to the common grape genome, *V. vinifera* version 2.1 [29], showed strong collinearity between the two assemblies, and chromosome 7 in the common grape assembly was shown to be split between chromosomes 7 and 20 in the ‘Carlos’ assembly, consistent with previous muscadine cultivar assemblies and linkage maps (Fig. 2) [19, 21, 22, 30].

**Table 2:** Quantitative statistics of collinearity between *Vitis rotundifolia* ‘Carlos’, *V. rotundifolia* ‘Trayshed’, and *V. rotundifolia* ‘Noble’ assemblies

‘Carlos’ Aligned to ‘Trayshed’			
Variation_type	Count	Length (‘Trayshed’)	Length (‘Carlos’)
Syntenic regions	551	337,011,859	334,838,553
Inversions	43	2,924,794	2,055,345
Translocations	562	7,624,712	7,764,274
Duplications (‘Trayshed’)	211	1,719,701	-
Duplications (‘Carlos’)	401	-	1,477,822
Not aligned (‘Trayshed’)	1272	52,190,426	-
Not aligned (‘Carlos’)	1504	-	58,644,910
‘Carlos’ Aligned to ‘Noble’			
Variation_type	Count	Length (‘Noble’)	Length (‘Carlos’)
Syntenic regions	563	301,890,417	297,062,683
Inversions	278	37,080,995	35,655,433
Translocations	427	11,422,729	11,474,627
Duplications (‘Noble’)	353	1,807,614	-
Duplications (‘Carlos’)	229	-	3,255,480
Not aligned (‘Noble’)	1580	43,540,564	-
Not aligned (‘Carlos’)	1345	-	59,119,165
‘Trayshed’ Aligned to ‘Noble’			
Variation_type	Count	Length (‘Noble’)	Length (‘Trayshed’)
Syntenic regions	624	294,764,948	294,233,443
Inversions	241	30,059,987	34,873,805
Translocations	608	16,742,580	16,733,441
Duplications (‘Noble’)	229	3,805,281	-
Duplications (‘Trayshed’)	715	-	4,612,284
Not aligned (‘Noble’)	1482	54,144,482	-
Not aligned (‘Trayshed’)	2001	-	51,185,359

Comparison of the three muscadine and the common grape genome annotation sets with GENESPACE, version 0.9.4, produced a synteny-based pan-genome (Table 3, Supplemental Table S3) [31]. GENESPACE predicted a total of 34,970 synteny-constrained orthogroups. Of these, 17,457 orthogroups contain at least one gene from each genome, representing the core *Vitis* gene set. Of these genes, 10,750 match exactly one gene from each genome, representing single-copy orthologs. The remaining 17,513 accessory orthogroups are missing gene(s) from at least one genome. 4,919 accessory orthogroups are represented by only genes from one genome, or “single-individual”. Of these, 4,321 have a single gene from a single genome, hereafter referred to as singleton genes, and 598 contained more than one gene from a single organism. Of the singletons, 426 were observed in ‘Carlos’, 607 were observed in ‘Noble’, 455 were observed in ‘Trayshed’, and 2,833 were observed in common grape. Annotation of the ‘Carlos’ singletons indicated the presence of RPV1 and RUN1, two muscadine disease-resistance genes [32], among other plant defense proteins (Supplemental Table S4), and annotation of the non-singleton, ‘Carlos’-only Orthogroups identified lignin-degrading proteins and germin-like proteins (Supplemental Table S4). The GENESPACE riparian synteny plot is consistent with the synteny plots produced by SyRi and RIdeogram (Supplemental Figure S3).

**Table 3:** ***Vitis*pan-genome information and statistics.** Four gene sets, three from muscadine genome assemblies ‘Carlos’, ‘Trayshed’, ‘Noble’ and one from *V. vinifera* were utilized. **“**Syntenic links” are regions of the genomes that share gene order. “Core” orthogroups refer to orthogroups with at least one gene member from each of the four gene sets. “Single-copy” orthogroups refer to core orthogroups with exactly one gene from each of the four gene sets. “Accessory” orthogroups refer to orthogroups where at least one gene set does not have an included gene. “Single-individual” orthogroups refer to orthogroups where genes from only a single gene set are present. “Singletons” refer to orthogroups containing only a single gene from a single gene set

Number of Initial Orthogroups	22,864
Number of Syntenic Links	1,084
Number of Synteny-Constrained Orthogroups	34,970
Number of Core *Vitis* Orthogroups	17,457 (49.9%)
Number of Single-Copy Orthogroups	10,750 (30.7%)
Number of Accessory *Vitis* Orthogroups	17,513 (50.2%)
Number of Single-Individual Orthogroups	4,919 (14.1%)
Number of Singletons	4,321 (12.4%)
Number of ‘Carlos’ Singletons	426 (1.2%)
Number of ‘Noble’ Singletons	607 (1.7%)
Number of ‘Trayshed’ Singletons	455 (1.3%)
Number of Common Grape Singletons	2,833 (8.1%)
Number of Non-Singleton Single-Species Orthogroups	598 (1.7%)

We ran RNAmmer, version 1.2 [28], on the assemblies for ‘Trayshed’, ‘Noble’, and common grape to compare predicted rDNA regions between other muscadine and grape assemblies (Fig. 1, Supplemental Tables S6-9). In the ‘Carlos’ assembly, recall from above there were two nucleolus organizing regions (NORs) found on chromosomes 15 and 17, and two 5S arrays were found on chromosome 17. In ‘Trayshed’, an NOR was only detectable on chromosome 17. In ‘Noble’, a single NOR on chromosome 15 was detected. We did not detect any complete NORs in the Version 2.1 assembly of the common grape genome (Fig. 2). Two 5S arrays along chromosome 17 were detected in all four assemblies.

**Fig. 3:**
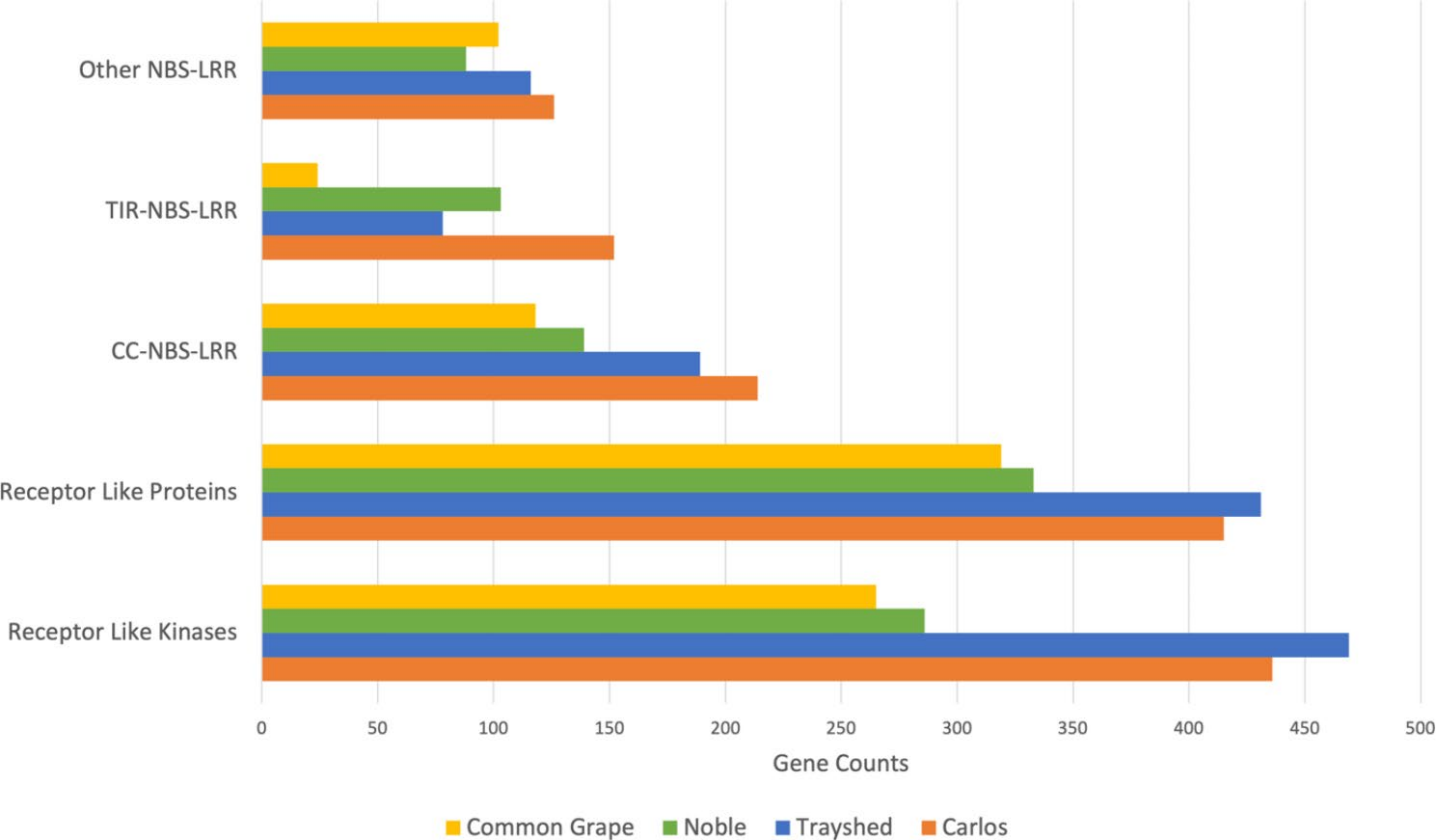
**Gene Counts of Resistance Gene Classes for Three Muscadine Cultivars and Common Grape.** Gene class was assigned based on the set of domains found in the gene (Supplemental Table S7)

We identified and classified predicted plant disease resistance-related genes (R-genes) in ‘Carlos’, ‘Trayshed’, ‘Noble’, and common grape to compare between tolerant, resistant, and susceptible individuals/species (Fig. 3). We first identified genes with R-gene relevant domains, including coiled-coil (CC), kinase, leucine rich repeat (LRR), nucleotide binding site (NBS), Toll/interleukin-1 receptor (TIR), and transmembrane helix domains. ‘Carlos’ contained a total of 3,522 genes containing domains associated with R-genes, a comparable number to ‘Trayshed’ (3,274), but higher than the number observed in ‘Noble’ (2,394) and common grape (2,332). These sets of genes were further categorized by domain combinations into common R-gene classes: receptor-like kinases (RLK), receptor-like proteins (RLP), TIR-NBS-LRRs (TNL), CC-NBS-LRRs (CNLs), and other NBS-LRRs (NLs). ‘Carlos’ and ‘Trayshed’ have similar gene counts in most classes, and are consistently higher than in ‘Noble’, except the TNL class. Common grape has the lowest number in all R-gene categories except NLs, where it has one gene more than ‘Noble’, yet still less than the other two muscadine cultivars. This reveals an overall trend of expansion of R-genes in muscadine grape species that is not present in common grape species. All four gene sets had a higher number of RLK and RLP class genes than other NBS-related classes (Supplemental Table S10).

We then identified orthogroups from GENESPACE that contained one or more R-gene candidates from ‘Carlos’. The 3,522 candidate R-genes were placed into 2,061 synteny-constrained orthogroups. Of these orthogroups, 250 were predicted to function as RLKs, 303 as RLPs, 84 as TNLs, 132 as CNLs, and 89 as other NLs. In comparing the number of genes in these orthogroups from ‘Carlos’ to those of common grape, we found that 518 of these orthogroups contain genes novel to ‘Carlos’, and 392 contain more genes in ‘Carlos’ compared to grape. The orthogroups unique to ‘Carlos’ contained 64 genes predicted to function as RLKs, 109 as RLPs, 50 as TNLs, 54 as CNLs, and 37 as other NLs. Of the 392 orthogroups with more ‘Carlos’ genes, 65 are predicted to function as RLKs, 166 as RLPs, 36 as TNLs, 87 as CNLs, and 57 as other NLs. For the remaining orthogroups, 244 contained more genes in grape than in ‘Carlos’, and 907 contained an equal number of genes between ‘Carlos’ and grape. These numbers support that a number of R-genes have either undergone expansions in muscadine or are unique to muscadine.

IV. Differential expression in response to Pierce’s Disease.

Four weeks after stem inoculation of *Xfsf* in ‘Carlos’ cultivar plants, RNA sequencing of leaf tissue revealed 234 differentially expressed (DE) genes compared to mock-inoculated control plants (Table 4, Supplemental Table S11). Of DE genes, 89 were up-regulated in infected ‘Carlos’ vines, and 145 were down-regulated. Annotation of these DE genes indicated a down-regulation of genes associated with lipid catabolism (mostly consisting of GDSL lipases), both up and down regulation of genes associated with oxidation-reduction, and an up-regulation of genes associated with ubiquitin, a proteolysis mediator associated with plant defense [33]. Genes predicted to function as lignin-degrading laccases were down-regulated, possibly indicating a strengthening of the cell wall through lignin fortification [34]. Genes associated with abscisic acid (ABA) biosynthesis, a pathway involved in drought resistance and stress response in plants [35], were mostly up-regulated, though some genes activated by ABA were down-regulated. Sixteen of the DE genes were found to have R-gene domains, 5 of which were assigned to the Kinase R-gene class, indicating a potential R-gene mediated plant defense response after inoculation with *Xfsf* (Tables 5, Supplemental Table S13). Of these candidate R-genes, 9 were down-regulated and 7 were up-regulated. VITMroCarlos_v1.3.g17552, an up-regulated gene with RLP domains, is predicted to function as Eix2, a receptor driving disease resistance in plants [36]. GhostKOALA, version 2.2 [37], analysis of the differentially expressed genes indicated an enrichment of pathways associated with metabolism, cell signaling, and genetic processing. Up-regulated genes were associated with the Toll and immune deficiency (IMD) signaling pathways, ubiquitin-mediated proteolysis, and terpenoid biosynthesis. The Toll and IMD pathways are consistent with up-regulation of R-genes containing Toll domains, and plant terpenoids have well-characterized roles in plant defense [38]. Down-regulated genes were associated with biosynthesis of phenylpropanoids and other plant secondary metabolites, plant-pathogen interactions, and cell cycle processes.

**Table 4:** **Differential expression results in*Vitis rotundifolia*‘Carlos’ and*Vitis vinifera.*** List of ‘Carlos’ DE genes with function is found in **Supplemental Table S11**

	Up-Regulated	Down-Regulated	Total	Representative Orthogroups
Muscadine (Control vs. Inoculated)	89	145	234	208
Grape (Control vs. Inoculated)	66	31	97	78
Grape (Inoculated vs. *wzy* KO)	206	12	218	165
Grape (Control vs. *wzy* KO)	124	11	135	102

**Table 5:** **Placement of candidate Resistance genes in Orthogroups.** RLP = Receptor-like protein; KIN = Kinase; CLK = Coiled-coil, leucine-rich repeat, kinase; RLK = Receptor-like kinase; CK = Coiled-coil, kinase; TNL = Toll/Interleukin receptor, nucleotide binding site, leucine-rich repeat. Log fold change from ‘Carlos’ RNASeq experiment, with positive values indicating up regulation in inoculated samples and vice versa. Grape and ‘Carlos’ Gene Count are the total number of genes from each found in the orthogroup. Shown in bold are those genes differentially expressed that are not present in common grape. Placement of R-genes in the pan-genome can be found in Supplemental Table S13

Class	Orthogroup	Candidate R-Gene	LogFoldChange	Grape Gene Count	‘Carlos’ Gene Count
CK	OG0002568	VITMroCarlos_v1.3.g25887	-2.1	2	2
	OG0009426	VITMroCarlos_v1.3.g15699	-1.8	1	1
CLK	OG0008270	VITMroCarlos_v1.3.g12650	0.9	5	5
KIN	OG0000032	VITMroCarlos_v1.3.g16025	3.6	29	32
	OG0000484	VITMroCarlos_v1.3.g10347	-1.3	2	3
	OG0005040	VITMroCarlos_v1.3.g19102	-2.2	1	2
	**OG0013253**	**VITMroCarlos_v1.3.g17633**	**-6.8**	**0**	**9**
		**VITMroCarlos_v1.3.g17647**	**-7.0**	**0**	**9**
	**OG0015829**	**VITMroCarlos_v1.3.g20953**	**6.5**	**0**	**5**
RLK	OG0000068	VITMroCarlos_v1.3.g21507	3.2	14	19
	OG0002084	VITMroCarlos_v1.3.g15393	-2.2	2	2
		VITMroCarlos_v1.3.g24514	-5.2	2	2
RLP	OG0000075	VITMroCarlos_v1.3.g01514	-1.1	9	6
	OG0005036	VITMroCarlos_v1.3.g17550	3.2	2	9
		VITMroCarlos_v1.3.g17552	3.3	2	9
TNL	OG0008267	VITMroCarlos_v1.3.g24911	3.2	1	8

In common grape, tolerance can be induced in otherwise susceptible cultivars by knockout of the *wzy* gene in *Xfsf*, which results in truncation of the complex, O-chain antigen that delays PAMP-triggered immunity (PTI) [17, 39]. It is currently unclear how muscadine grapes, which have greater tolerance to wild type *Xfsf*, are able to bypass the O-chain antigen, or if it has an alternative method of tolerance. To gain insight into the possible differences or overlaps of these two grape species to *Xsfs*, we examined the overlap of the gene orthogroups between our dataset and data from Rapicavoli et al., 2018. Rapicavoli et al. 2018 reported an RNASeq experiment with inoculation of ‘Cabernet Sauvignon’ grapes with wild type *WZY Xfsf*, mutant *wzy Xfsf*, or control buffer. RNA sequencing in tissue both local and systemic to the site of inoculation spanned collection times ranging from hours to 4 weeks after inoculation [17]. We selected data that mirrored our experimental design, i.e. systemic tissue collected four weeks after inoculation, for comparison.

**Fig. 4:**
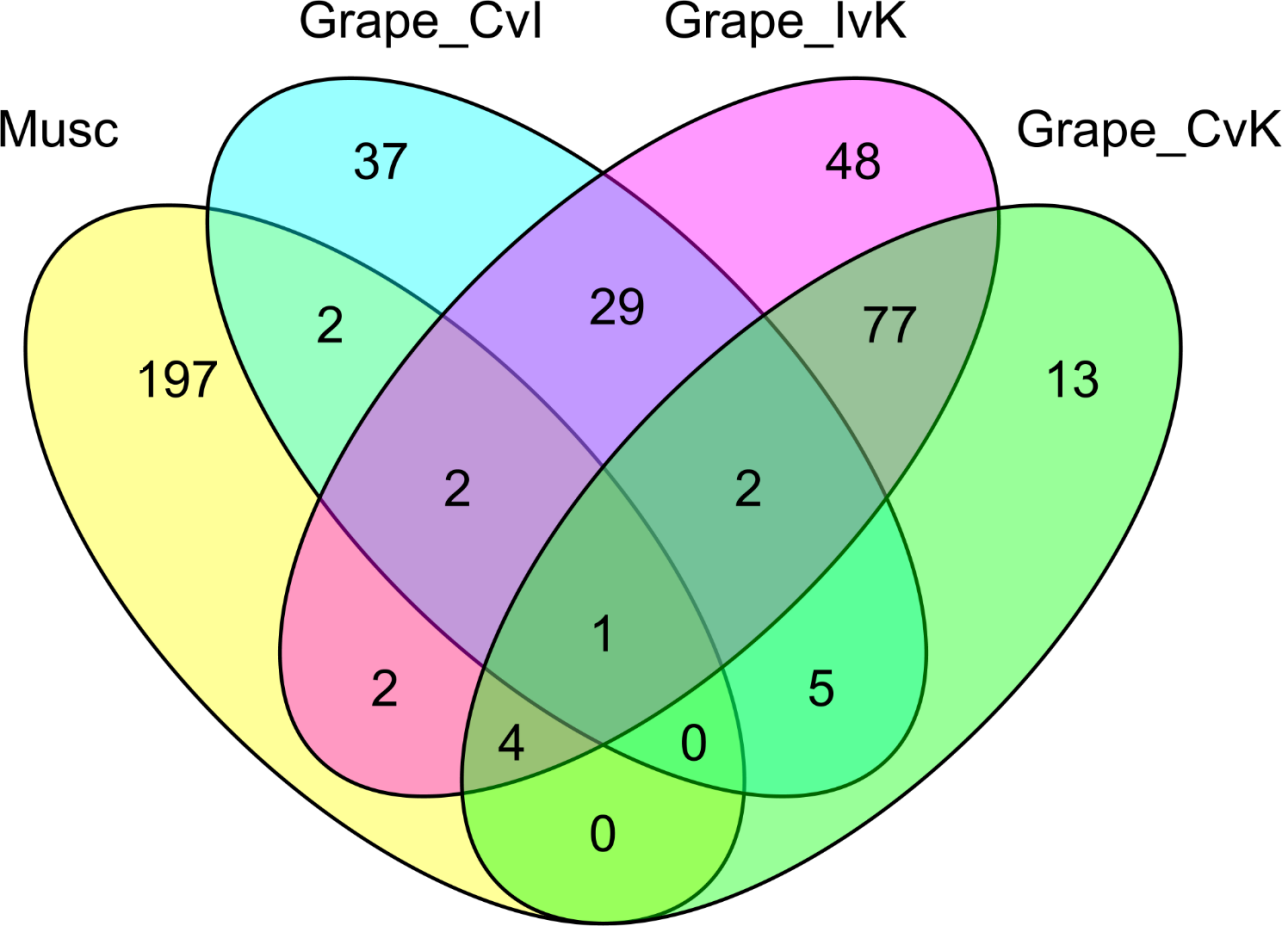
**Overlap of orthogroups associated with differentially expressed genes in*Vitis rotundifolia*‘Carlos’ and*Vitis vinifera***. Orthogroups contained one or more genes that were differentially expressed in the comparison. “Musc” = Muscadine Control vs. Inoculated. “Grape_CvI” = Grape Control vs. Inoculated (Wild-type). “Grape_IvK” = Grape Inoculated (Wild-type) vs. Inoculated (*wzy* Knockout). “Grape_CvK” = Grape Control vs. Inoculated (*wzy* Knockout)

Genes were compared between species by orthogroups, with each orthogroup representing a common ancestral grape gene for all orthogroup members. The 25,556 orthogroups identified by OrthoFinder analysis were filtered for orthogroups containing the genes of interest - including orthogroups containing two or more differentially expressed genes - and an overlap between all four conditions was determined (Fig. 4). Most orthogroups containing muscadine DE genes were unique to the muscadine response, with any grape gene members not reported as differentially expressed. Only 1 orthogroup, OG0000266, shared genes that were differentially expressed (all up-regulated) in all four comparisons (Table 6). Functional annotation of the muscadine DE gene defined it as a Dirigent protein, a plant defense gene associated with lignin biosynthesis [40, 41]. Four orthogroups contained genes that were differentially expressed in the three comparisons associated with greater tolerance to PD. Functional annotation of these orthogroups of interest identified more genes associated with plant pathogen response, including Snakin-1 and an auxin-binding protein (Table 6). Half of these genes were up-regulated in muscadine, and the other half were down-regulated. A full list of orthogroups, including those containing DE muscadine genes, is available in Supplemental Table S12.

**Table 6:** **Functional annotation of orthogroups with differentially expressed (DE) genes in muscadine and grape.** Functional annotation was performed using EnTAP version 0.10.8. The listed Gene IDs include only the genes that are differentially expressed. “Musc” indicates the Muscadine control vs. inoculated comparison, “Grape IvK” indicates the wild-type *X. fastidiosa* subsp. *fastidiosa* inoculated Grape vs. *wzy* knockout strain inoculated Grape comparison, and “Grape CvK” indicates the Grape control vs. *wzy* knockout strain inoculated Grape comparison

Orthogroup ID	*V. vinifera* DE Gene IDs	*V. rotundifolia* (Carlos) DE Gene IDs	DE in Comparisons	Protein Function	DE in Infected Muscadine
OG0000266	VIT_206s0004g01000.1, VIT_206s0004g01015.1	VITMroCarlos_v1.3.g23332	All	Dirigent Protein, Disease resistance response protein	Up
OG0000539	VIT_218s0001g14810.1	VITMroCarlos_v1.3.g24572	Musc, Grape IvK, and Grape CvK	Lipase_GDSL domain-containing proteinGDSL esterase lipase	Down
OG0000585	VIT_207s0129g00580.1	VITMroCarlos_v1.3.g18058	Musc, Grape IvK, and Grape CvK	Snakin-1, GASA Family protein	Down
OG0001153	VIT_212s0034g01890.1, VIT_212s0034g01930.1, VIT_212s0034g01950.1, VIT_212s0034g01970.1	VITMroCarlos_v1.3.g00557	Musc, Grape IvK, and Grape CvK	Globulin seed storage protein 2	Up
OG0011935	VIT_209s0002g01320.1	VITMroCarlos_v1.3.g13207	Musc, Grape IvK, and Grape CvK	Germin-like protein, auxin-binding protein ABP19a-like	Up

The 16 differentially expressed R-gene candidates from the ‘Carlos’ DE gene set were placed into 13 orthogroups in this analysis. While most of these orthogroups contained genes from the common grape assembly, only the muscadine genes were differentially expressed in response to infection with *Xfsf* (Table 5, Supplemental Table S13). One orthogroup contained more genes in common grapes compared to ‘Carlos’, and 5 contained an equal number of genes between the two species. The remaining orthogroups contained more genes from ‘Carlos’ than in common grapes. Of these, 2 orthogroups - OG0013253 and OG0015829 - contained no genes from the common grape annotation, indicating proteins novel to the muscadine annotation (Table 5, **bold; Supplemental Table S13, bold**). The R-genes in OG0013253 are located on Chromosome 16, and the gene in OG0015829 is located on Chromosome 10. These R-genes are predicted to function as kinases - from both the annotation results and the R-gene analysis - with the R-genes of OG0013253 being down-regulated in response to *Xfsf* and the R-gene of OG0015829 being up-regulated. Another orthogroup, OG0005036, contained VITMroCarlos_v1.3.g17552, previously established as a predicted plant defense gene. Orthogroup analysis indicates an expansion in this family in ‘Carlos’ compared to common grapes, supporting an R-gene expansion.

## Discussion

Pierce’s Disease, caused by the gram-negative bacteria *Xylella fastidiosa subsp. fastidiosa*, represents a limiting factor in the production of Californian common grapes, yet is inconsequential to wild and commercial muscadine grapes grown as a native food crop in the Southeast. While current understanding of muscadine’s mechanisms of tolerance and resistance is limited, current research suggests that muscadine grapes produce novel proteins that improve host tolerance [42] or produce tyloses to prevent spread of the bacteria [13]. A better understanding of genetic and molecular resistance mechanisms will allow researchers to improve tolerance in common grape varieties through either gene introgression or selection of traits through breeding. To that end, we have worked to expand our existing knowledge of the muscadine genome and PD tolerance by providing a genome assembly and *Xfsf* response transcriptome for the tolerant cultivar ‘Carlos’.

The ‘Carlos’ muscadine genome assembly is the third public reference genome from muscadine, following the ‘Trayshed’ and ‘Noble’ reference genomes [19, 22]. Using the ‘Trayshed’ assembly as a guide, our assembly anchored over 99% of sequenced bases into 20 chromosome-level scaffolds. By default, this method resulted in chromosome-level scaffolds, but still containing a number of gap regions. We were able to resolve a number of the gaps using raw PacBio reads, resulting in an assembly with a low percentage of uncalled bases. Structural comparison of the ‘Carlos’ assembly to the ‘Trayshed’ assembly showed that, because of the high-quality contig assembly, we were able to identify structural variants between the two assemblies [19]. Comparison of ‘Carlos’ with the ‘Noble’ assembly is consistent with the collinear analysis from the ‘Noble’ paper [22], finding most structural variants previously observed between ‘Noble’ and ‘Trayshed’ were also identified when comparing ‘Carlos’ and ‘Noble’. Due to the ‘Noble’ assembly utilizing a combination Illumina and PacBio CLR reads - as opposed to our PacBio CLR-only approach -it is likely that some of these structural variations may be artifacts of a lower contiguity assembly in ‘Noble’. Supporting this, the contig N50 of the ‘Noble’ assembly is 105.8 KB, while the contig N50 for the ‘Carlos’ assembly is 11.3 MB. Recent developments in long-read sequencing, in particular the PacBio HiFi technology, have resulted in more accurate assemblies using long-reads alone than either hybrid- or short-read assemblies only [43, 44]. As accurate long-read sequencing becomes more common, future genome assemblies will provide more accurate insights into true structural variation, as genome assembly quality is a key factor in these pan-genome type comparisons [45].

Ribosomal gene arrays (rDNA arrays) are fundamental parts of genome architecture with high tandem copy numbers. In plants, the two array types − 5 and 35 S (18 S-5.8 S-26 S) - can occur in one or a few locations in the genome. In *Vitis* species, rDNA arrays have been used to profile chromosome architecture and to infer phylogenies, and results of the available assemblies indicate the number and length of both array types varies within *V. vinifera* cultivars and across *Vitis* species [46, 47]. These arrays represent a challenge to most assembly algorithms, as their total length may exceed even long read sequencing reads. We compared placement of rDNA arrays along the ‘Carlos’ assembly to those of existing muscadine and grape cultivars. The two 5 S rDNA locations on ‘Carlos’ chromosome 17 were shared with the ‘Trayshed’ and ‘Noble’ muscadine assemblies, but each of the existing genomes missed one of the two 35 S arrays we identified in ‘Carlos’. Often, rDNA array presence was associated with large structural variations (Fig. 1). For example, the NOR along Chromosome 15 in ‘Carlos’ is associated with a large inversion between the ‘Carlos’ and ‘Noble’ assemblies. These differences in rDNA array locations between otherwise similar genomes highlight the difficulty in capturing these repetitive regions in genome assemblies. Similarly, we found that NORs could not be reliably found in the common grapevine version 2.1 genome. Further cytogenetic work in these specific cultivars is needed to confirm the actual 5S and 35S loci locations, confirm nearby structural variants (or indicate errors in assembly), and tie the reference genomes back to the chromosomal karyotypes.

There are over 100 cultivars of muscadine available in and beyond its native range [48]. Recent studies have found substantial genetic diversity within muscadine cultivars [49, 50]. Attempting to use a single one of the three available genomes to characterize every cultivar will result in gaps in knowledge due to the phenotypic and genotypic differences between each cultivar [23, 51]. The solution is to further develop resources for these three cultivars - as well as additional cultivars - to develop a pan-genome. Pan-genomes cover the complete gene content of multiple cultivars of a single species, or multiple species of a closely-related genus. The pan-genome approach has been applied with great success to common crops such as maize, tomato, and rice [52–55]. In the context of *Vitis* species, pan-genome data can be used to better understand the mechanisms driving disease resistance, drought tolerance, and other traits of interest. For this study, we produced a foundational muscadine pan-genome consisting of the three muscadine cultivar annotations, as well as the common grape annotation. Of the 34,970 synteny-constrained orthogroups, almost half contain at least one gene from all four annotations, representing the “core” gene set across all four genomes. The other orthogroups represent “accessory” genes, including 4,919 orthogroups containing only genes from a single genome. As more grape, muscadine, and other *Vitis* genomes continue to be produced, we will add new *Vitis* genomes to our pan-genome, and our understanding of what genes are currently considered “core” and “accessory” to evolve.

In common grapes, pattern-associated molecular pattern (PAMP)-triggered immunity (PTI) is delayed by a long O-chain antigen associated with lipopolysaccharides (LPSs) in *Xfsf*. Knock-out of the *wzy* gene results in grapevines mounting a faster immune response and having greater tolerance to infection [17]. By contrast, muscadine cultivars range from tolerant to resistant against the bacteria, indicating that it is successfully detecting the pathogen by overcoming the O-chain antigen or utilizing another mechanism of detection. Plant resistance (R) genes are of particular importance to pathogen detection and response signaling [56], and the immunity observed in muscadine cultivars could be attributed to an expanded suite of R-genes compared to the R-genes of common grapes [32]. The ‘Trayshed’ assembly paper reported an expansion of R genes in muscadine grapes compared to common grapes [19]. Analysis of R-genes in the ‘Carlos’ assembly supports this expansion, with more R-genes identified in the ‘Carlos’ assembly than in ‘Trayshed’. The Trayshed genome paper described an expansion of Toll/Interleukin-1 Receptor-like Nucleotide-Binding Site Leucine-Rich Repeat (TIR-NBS-LRR) genes compared to the common grape. R-gene analysis of our gene annotation supported this expansion and indicated a nearly two-fold expansion of TIR-NBS-LRR genes in ‘Carlos’ compared to ‘Trayshed’. The ‘Noble’ assembly appears to have a contraction in most R-genes compared to other muscadine cultivars, though this may be the result of a different annotation strategy and deserves further exploration. Despite the overall lower number of R-genes compared to the other cultivars, ‘Noble’ had more proteins containing TIR-NBS-LRR domains compared to the ‘Trayshed’ assembly. The disparity in R-genes identified in Noble may be attributed to a lower quality assembly. An enriched set of R-genes in muscadine grape genomes supports the notion of improved disease resistance mechanisms in muscadine compared to the common grape. A recent GWAS (genome wide association study) in V. arizonica identified eight genomic regions associated with PD resistance, many containing R genes [57]. The authors attempted to extend their results to an additional six wild *Vitis* species using a k-mer bioinformatics approach. They identified a greater overlap of resistance-associated k-mers between closely-related species than between more distant species, suggesting that the resistance trait evolved independently and more than once [57]. Further work will be needed to tease out candidates in *V. rotundifolia* associated with improved response to infection with *Xfsf*.

The results of our gene expression analysis in ‘Carlos’, reveal that abiotic stress response, particularly drought tolerance, were up-regulated in ‘Carlos’ inoculated with *Xfsf*, with a few up-regulated genes associated with defense. Of these defense genes, we observed a candidate gene, VITMroCarlos_v1.3.g17552, that showed similarity to EIX2, a receptor-like protein that confers resistance to a fungal pathogen of tomato after detecting an elicitor protein [36]. While LPSs are well-characterized elicitors in common grapes, they are not the only PAMP that can induce PTI [58]. Orthogroup placement of this gene indicates an expansion in this family compared to grape, which could point to an expanded suite of PAMP response elements in muscadine compared to common grape (Table 5). It is important to note that, instead of samples collected from the site of inoculation, our sequencing data measured systemic responses to *Xfsf* inoculation. These results, therefore, offer no conclusions on parenchymal cell response in the xylem, where initial detection of *Xfsf* occurs. Further research will be needed to fully characterize the muscadine response to inoculation with *Xfsf*. In particular, it will need to be determined if the tolerance observed in ‘Carlos’, as opposed to resistance in other cultivars, can be attributed to a different immune response, or if it is the result of greater drought tolerance compared to common grapes.

To contextualize how these observed genes overlap with the common grape response to *Xfsf*, we compared the gene expression of ‘Carlos’ muscadines inoculated with *Xfsf* to grapes inoculated with two strains: a wild-type strain and a *wzy*-knockout strain [17]. Rather than attempting to map reads from one organism to the genome of another, we utilized OrthoFinder to identify genes with a common ancestor of both species. Of the 208 orthogroups containing differentially expressed genes in muscadine, 197 were unique to the muscadine response to PD. Of note, 19 of these orthogroups contained no genes from the common grape annotation, suggesting them as interesting candidates for follow up study. For the remaining orthogroups with both muscadine and common grape members, unique changes in gene sequence or expression patterns may have emerged after the organisms diverged evolutionarily to drive a unique host response to PD.

Despite the variation in host susceptibility, some overlaps in DE genes between muscadine and common grape were found. One orthogroup contained genes that were differentially expressed across all four conditions, and four contained genes that were differentially expressed among all PD-tolerant responses (Fig. 4). The single orthogroup representing all four conditions, OG0000266, was annotated as a Dirigent (DIR) protein. Among other important functions, plant (DIR) proteins play a role in plant defense response by modulating cell wall metabolism through lignin accumulation [40]. In muscadine, this gene was up-regulated in response to inoculation with *Xfsf*. The four orthogroups associated with PD-tolerant responses were also functionally annotated as genes associated with plant pathogen responses [59–62]. Among these, the muscadine gene coding for a GDSL lipase was down-regulated, which is consistent with the observed DEGs. While GDSL lipases can play a role in plant defenses [63], some plant systems see improved host resistance when GDSL lipases are silenced [61]. While there is overlap in genes differentially expressed in muscadines inoculated with *X. fastidiosa* subsp. *fastidiosa*, our main conclusion is that most differentially expressed genes were uniquely expressed in muscadine, supporting a unique host response not found in common grapes. While hybrids between common grapes and muscadine grapes have been produced, these efforts are made complicated by a chromosome-fusion event in common grapes. By identifying specific genes with high association to PD-resistance in muscadine, researchers will be better able to focus their efforts on candidate resistance genes.

## Conclusions

Our efforts in this study have provided three major outcomes to advance the understanding of the muscadine grape. First, our ‘Carlos’ cultivar assembly adds to the existing genome reference sequences and gene annotation sets available for muscadine grapes. Second, our *Vitis* pan-genome provides a new resource for comparative genomics between muscadine cultivars and those of the common grape, while allowing room for expansion. Third, we provide a comparison of differentially expressed genes that are informative in comparing the immune response between muscadine and common grapes. There is more work to be done to fully characterize the muscadine response to Pierce’s disease, from an expanded time course at different times and locations after infection to host and pathogen knockout experiments. Our results have provided new resources to expand this understanding and provided a framework for future sequencing efforts.

## Materials and methods

I. Plant sampling - DNA sequencing.

The muscadine grape clone ‘US19-33’, a self-pollinated accession of *V*. *rotundifolia* Michx., (2*n* = 2*x* = 40) cultivar ‘Carlos’ [14], maintained at the USDA ARS Southern Horticultural Research Laboratory in Poplarville, MS was selected for genomic DNA (gDNA) extraction (30.8402° N, 89.5342° W). Young leaves were collected from a single plant of clone ‘US19-33’ and used for gDNA extraction using a modified hexadecyltrimethylammonium bromide (CTAB) protocol (Nishiyama et al. 2021). Isolated gDNA was quantified with a Nanodrop 2000 spectrophotometer and a Qubit dsDNA HS assay kit (ThermoFisher Scientific, Waltham, MA), and gDNA quality was assessed using an Agilent 2100 Bioanalyzer (Agilent Tech, Santa Clara, CA, USA). Ultimately, gDNA libraries for Illumina short-read sequencing were prepared, and paired-end sequencing (2 × 150 bp, 30× coverage) was performed on the Illumina NovaSeq 6000 sequencing platform (Illumina, San Diego, CA, USA).

To isolate high molecular weight (HMW) DNA suitable for PacBio long-read sequencing, young expanding leaves were dark-treated for 48 h prior to harvest and flash frozen in liquid nitrogen prior to gDNA extraction. Nuclei were isolated using the Bionano Prep Plant Tissue DNA Isolation kit (Bionano Genomics, SanDiego, CA, USA). Subsequently, HMW DNA was extracted from the nuclei using the Circulomics Nanobind Plant Nuclei Big DNA kit (Pacific Biosciences, Menlo Park, CA, USA). HMW DNA was used to construct libraries following the manufacturer’s protocol and sequenced using the continuous long read (CLR) protocol on the Pacific Biosciences (PacBio) sequencing platform (Pacific Bioscience, Menlo Park, CA, USA).

II. Plant sampling - RNA sequencing.

A total of 8 plantlets from tissue culture propagated muscadine grape cultivar ‘Carlos’ were transferred from PlantMedia Magenta boxes (PlantMedia, Dublin, OH, USA) into 1-gallon plastic pots containing a mixture of pine bark mulch and sand (1:1, v/v) with a pH of 5.6. Stems were needle-inoculated approximately 7 cm above the media surface with 10 µl of succinate-citrate buffer containing 10^6^ cells of *X. fastidiosa* subsp. *fastidiosa* (*Xfsf*) strain NOB1 [64]. Control plants were mock inoculated with 10 µl of succinate-citrate buffer. Plants were stored inside an incubator maintained at the following conditions: 25° C temperatures during 16 h photoperiods of 154 µmol m^− 2^s^− 1^ photon flux density, 22° C temperatures during 8 h skotoperiods, and 65% consistent relative humidity.

Approximately 0.1 g of leaf tissues were collected 28 days post-inoculation and used for DNA and RNA extraction. Total genomic DNA was extracted using a Cetyltrimethyl ammonium bromide (CTAB) method and used for the detection of *Xfsf* using RST31/RST33 primer pairs as described by Minsavage et al. 1994[65] [65]. Total RNA was isolated from leaf tissues collected from control and PD-inoculated plants using the Spectrum plant total RNA kit protocol (Sigma-Aldrich, MO, USA). RNA quantity and purity were assessed with a Nanodrop 2000 spectrophotometer and a Qubit 2.0 Fluorometer (Thermofisher, Waltham, MA, USA), respectively, and RNA quality was evaluated using an Agilent 2100 Bioanalyzer (Agilent Tech, Santa Clara, CA, USA). RNA sequencing libraries were constructed using the NEBNextTM II Directional RNA library prep kit, and sequencing was performed on the Illumina NovoSeq 6000 platform (Illumina, San Diego, CA, USA).

III. Genome scaffolding and assembly.

CLR reads were initially assembled into contigs using MECAT2 version 2019.03.04 [66], which implemented CANU version 1.9 [67]. Resulting contigs underwent one round of polishing with Arrow (Pacific BioSciences SMRT Tools Reference Guide, 2019), followed by additional polishing with Pilon [68]. Circular contigs, which were labeled in the output of CANU [67]v. 1.9, were removed from the assembly. We labeled this initial assembly version 1.1.

Following initial contig-joining, RagTag version 1.0.1 [69] was used to align the ‘Carlos’ contigs to the previously published ‘Trayshed’ assembly (focusing on haplotype 1). This produced a chromosome-level assembly with gap regions, labeled assembly version 1.2. These gaps were resolved with raw PacBio reads by running TGS-GapCloser, version 1.1.1 [70], producing our final assembly, labeled version 1.3. Benchmarking Universal Single-Copy Orthologs (BUSCO), version 5.2.2, was run to assess the completeness of the ‘Carlos’ cultivar assembly [25]. Statistical analysis of the scaffolds was performed using the BBtools “stats” command [71]. Repetitive regions were identified using RepeatModeler [72] and softmasked using RepeatMasker [73]. RNA-associated repetitive sequences were left unmasked.

IV. Gene annotation.

Gene Model Maker (GeMoMa) version 1.8 was used to predict the ‘Carlos’ gene annotation using the existing annotation for the ‘Trayshed’ haplotype 1 genome assembly [26]. GeMoMa includes an option to enhance the lift-over by incorporating RNASeq read alignment files, which we used to include the PD-inoculation transcriptome data in this analysis. Thereafter, input RNASeq reads were trimmed with Skewer version 0.2.2 [74], and trimmed reads were aligned to the ‘Carlos’ assembly with STAR version 2.7.9a [75]. All alignment files were merged into a single file using the Samtools version 1.10 “merge” command [76]. In addition to including the alignment data, GeMoMa was run with options to include untranslated regions (UTRs) and other features from the ‘Trayshed’ annotation. Identified genes were renamed, and gFACs version 1.1.2 was run without any filtering parameters to produce updated coding sequence (CDS) and amino acid (AA) FASTA files [77]. Non-coding RNA features such as rRNA and tRNA were annotated using RNAmmer version 1.2 [28] and tRNAScan version 2.0.9 [27, 78].

V. Comparative genomics of ‘Carlos’ to other genomes.

The ‘Carlos’ chromosomes were aligned to the ‘Trayshed’ haplotype 1 chromosomes and the ‘Noble’ chromosomes using MiniMap2 version 2.24 with default settings [79]. The resulting alignment files were run through synteny and rearrangement identifier (SyRI), version 1.6.3, to identify any structural rearrangements [80]. The resulting output was placed into SyRI’s sister program PlotSR, version 0.5.4, to visualize all structural rearrangements between ‘Carlos’ and the aforementioned ‘Noble’ and ‘Trayshed’ genomes [81]. In-depth gene annotation comparison and initial pan-genome construction was performed using GENESPACE, version 0.9.4 [31]. The common grape (*Vitis vinifera*), version 2.1, annotation was included in the GENESPACE analysis [29]. A custom script was developed to obtain statistics from the GENESPACE results [82]. Plant disease resistance-related genes (R-genes) were identified in these four genomes using the Disease Resistance Analysis and Gene Orthology (DRAGO2) pipeline [83].

Due to grape and muscadine having a different total number of chromosomes, SyRI could not be run to compare assembly structure. Instead, Orthofinder version 2.3.12 was used to identify orthologs between ‘Carlos’ muscadine and common grape [84]. Protein sets from a total of 7 other species, as well as the ‘Trayshed’ haplotype 1 annotation, were included as outgroups to enhance OrthoFinder phylogenetic results based on the suggested best practices in the OrthoFinder documentation [85]. Multiple sequence alignment (MSA) mode was selected using MAFFT version 7.467 to obtain MSA files [86]. Orthogroups with a single gene member from both ‘Carlos’ and common grape were selected [87, 88], and the related orthologous links were plotted using RIdeogram version 0.2.2 to visualize synteny between the genomes [89].

VI. Differential expression analysis.

The ‘Carlos’ RNASeq data was trimmed using Skewer, version 0.2.2, with identical parameters to those used in the gene annotation Sect. [74]. STAR, version 2.7.9a [75], was rerun for the muscadine samples against the ‘Carlos’ genome with gene annotation included. Following alignment, gene features were associated with read counts using HTSeq-Count version 0.13.5 [90]. Differential expression was determined using DESeq2 version 1.26.0 analysis of the read count data [91]. Expression was compared between the control samples and the inoculated samples. Significantly differentially expressed genes were called with an adjusted *p*-value of 0.05. DRAGO2 was rerun on the differentially expressed genes to identify candidate R-genes.

Common grapevine RNASeq data were obtained from a previous study that confirmed the effect of *wzy* knockout in *Xfsf*, strain *Temecula1*, on host response (NCBI accession: PRJNA345471) [17]. Due to the difference in strains, we compared the existing annotations of both strains to confirm that no significant structural variation occurred in *Wzy* between the strains. To match the conditions of our ‘Carlos’ RNASeq data, samples were only included if collected 4 weeks after inoculation from petioles. A total of 9 samples were analyzed: 3 representing control, 3 representing infection with wild-type *Temecula1* strain *Xfsf*, and 3 representing infection with mutant *wzy* knockout *Temecula1* strain *Xfsf*. For both sets of samples, Skewer, version 0.2.2, was run using identical parameters to those used in the gene annotation section. The same version and parameters of STAR were run to align the reads with the *V. vinifera* genome, version 2.1. For the common grape data, expression was compared between the control and inoculated samples, the inoculated and mutant samples, and the control and mutant samples (SRR4345376-SRR4345378, SRR4345409-SRR4345411, and SRR4345433-SRR4345435) using the same adjusted p-value to call differentially expressed genes.

To compare the expression of genes in ‘Carlos’ muscadine to common grape, the results of the earlier OrthoFinder run were used. Orthogroups containing significantly differentially expressed genes from analyses were identified. Functional gene annotation of ‘Carlos’ was determined using EnTAP version 0.10.8 [92], which assigned functions based on sequence similarity, Gene Ontology (GO) term assignment, and Kyoto Encyclopedia of Genes and Genomes (KEGG) pathway annotation. The Muscadine proteins of interest were also functionally annotated using KEGG’s BlastKOALA and GhostKOALA functions [37]. Impacted pathways were determined by running KEGG separately on up- and down-regulated genes.

## Electronic supplementary material

Below is the link to the electronic supplementary material.

Supplemental Figure S1: Foliage and pollinated flowers of Vitis rotundifolia cv. ‘Carlos’ vines. Picture taken in Poplarville, MS

Supplemental Figure S2: Pierce’s Disease symptoms in Vitis vinifera vines. Picture taken in Poplarville, MS

Supplemental Figure S3: Riparian plot of synteny between the 4 gene sets of the Vitis pangenome. Riparian plot generated using GENESPACE version 0.9.4

Supplemental Tables S1-S13: Summary of Early Forms of the Vitis rotundifolia ‘Carlos’ genome assembly. “Reference-Guided Scaffolds” refers to assembly before gap-closing

## Data Availability

The ‘Carlos’ raw PacBio reads and RNA reads are available in NCBI under Bioproject PRJNA935741. The genome assembly and annotation are available in DOI 10.5281/zenodo.7944874.

## Abbreviations

AA: Amino Acid
BUSCO: Benchmarking Universal Single-Copy Ortholog
CC: Coiled-Coil Domain
CDS: Coding Sequence
CLR: Continuous Long Read
CNL: CC-NBS-LRR
CTAB: Cetyltrimethyl ammonium bromide
DE: Differentially Expressed
gDNA: Genomic DNA.
GO: Gene Ontology
GWAS: Genome-Wide Association Study
KEGG: Kyoto Encyclopedia of Genes and Genomes
LRR: Leucine-Rich Repeat
MGSE: Muscadine Grape Skin Extracts
MSA: Multiple Sequence Alignment
NBS: Nucleotide-Binding Site
NL: NBS-LRR
NOR: Nucleolus Organizing Region
PAMP: Pathogen-Associated Molecular Pattern
PD: Pierce’s Disease
PTI: PAMP-triggered Immunity
QTN: Quantitative Trait Nucleotides
R-gene: Resistance Gene
RLK: Receptor-Like Kinase
RLP: Receptor-Like Protein
TIR: Toll/Interleukin Receptor-1
*Xfsf*: *Xylella fastidiosa* subsp. *fastidiosa*
