# Supplementary figures and images for: Long-read, chromosome-scale assembly of Vitis rotundifolia cv. Carlos and its unique resistance to Xylella fastidiosa subsp. fastidiosa
Source: BMC Genomics. 2023 Jul 20;24:409. doi: 10.1186/s12864-023-09514-y (PMC10357881; doi:10.1186/s12864-023-09514-y)

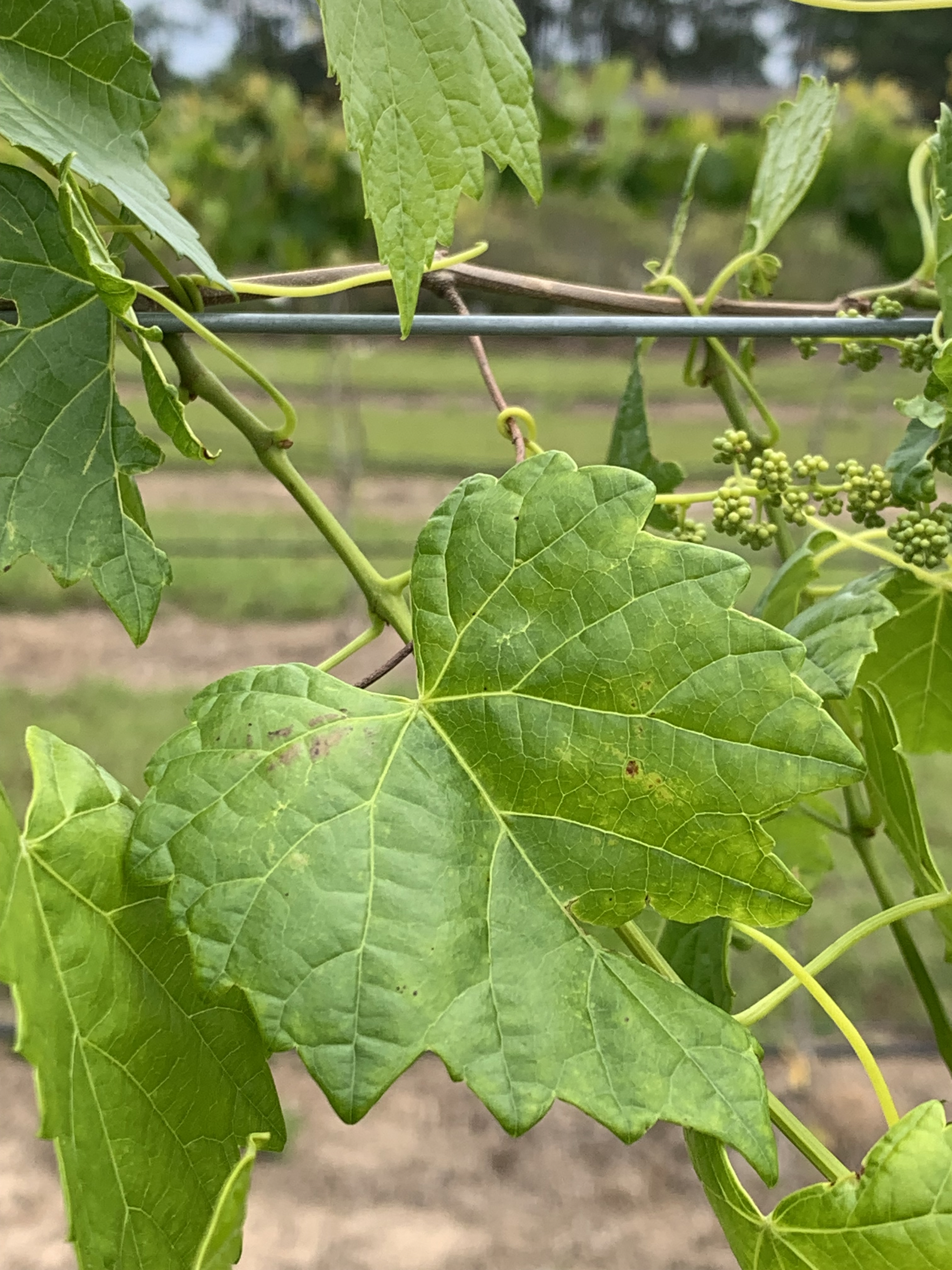

Supplement: Supplementary file 1 — Supplemental Figure S1: Foliage and pollinated flowers of Vitis rotundifolia cv. ‘Carlos’ vines. Picture taken in Poplarville, MS [file 12864_2023_9514_MOESM1_ESM.jpeg]

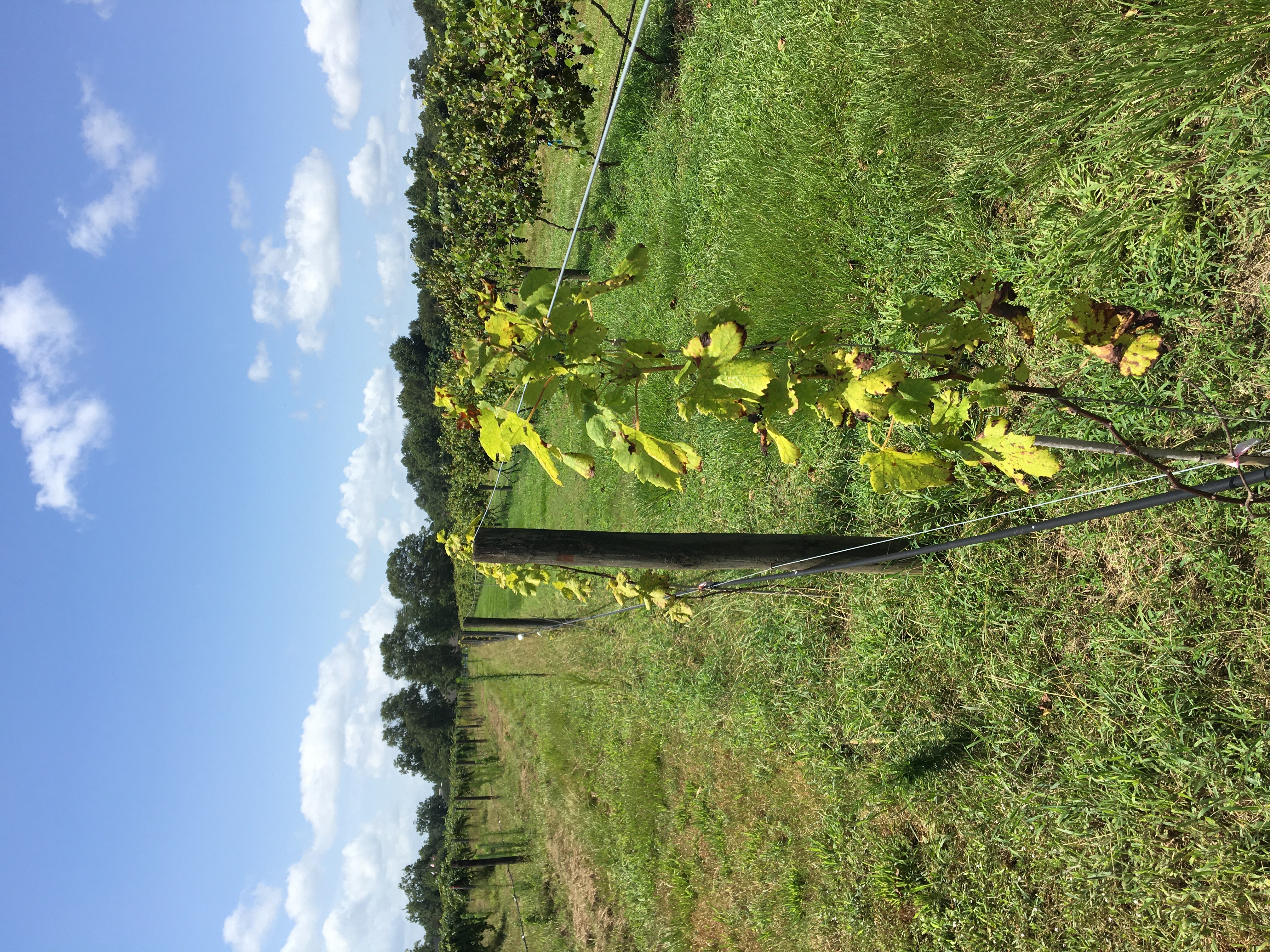

Supplement: Supplementary file 2 — Supplemental Figure S2: Pierce’s Disease symptoms in Vitis vinifera vines. Picture taken in Poplarville, MS [file 12864_2023_9514_MOESM2_ESM.jpg]

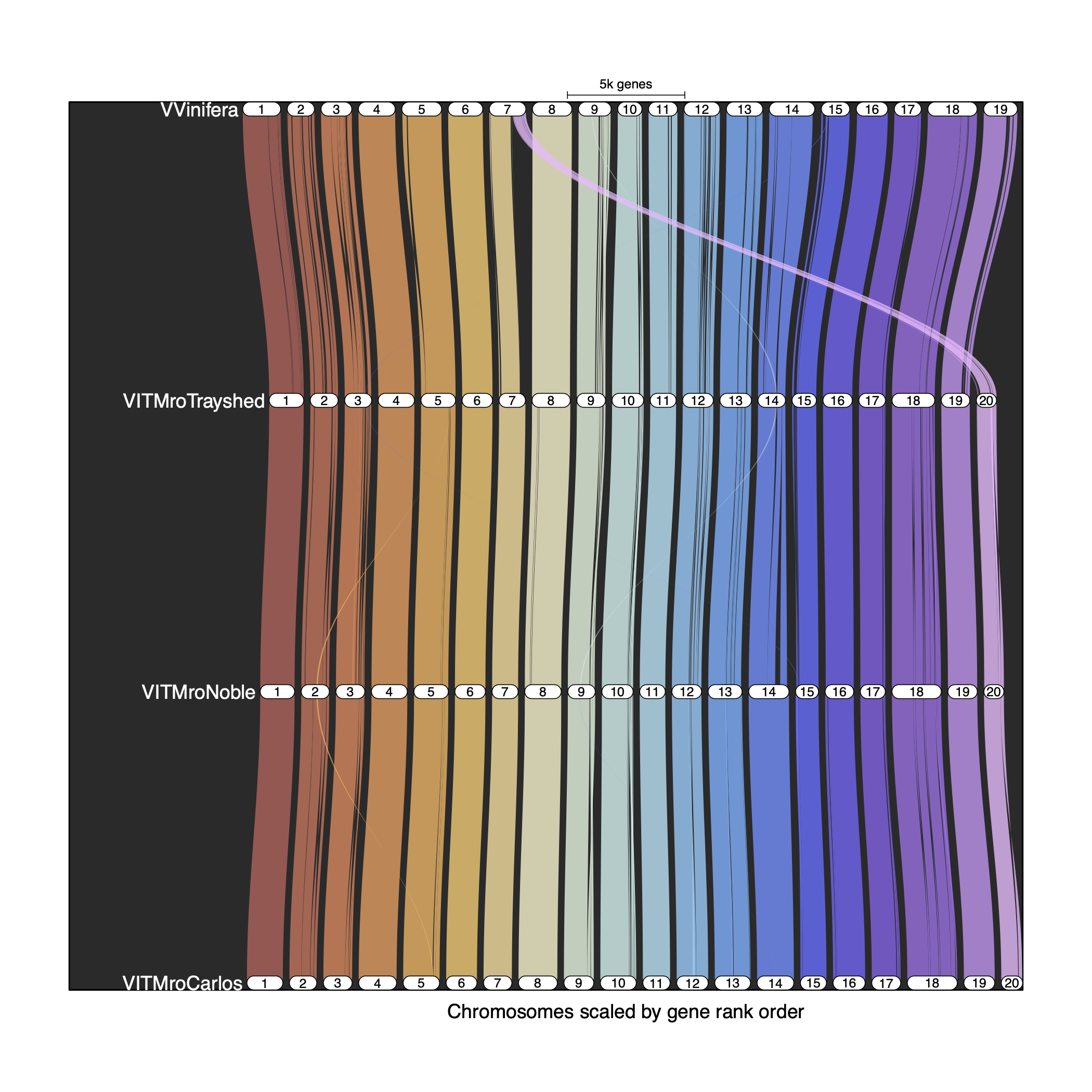

Supplement: Supplementary file 3 — Supplemental Figure S3: Riparian plot of synteny between the 4 gene sets of the Vitis pangenome. Riparian plot generated using GENESPACE version 0.9.4 [file 12864_2023_9514_MOESM3_ESM.png]
